# Measuring Theory of Mind in Adults with Autism Spectrum Disorder

**DOI:** 10.1007/s10803-017-3080-x

**Published:** 2017-03-09

**Authors:** Neil Brewer, Robyn L. Young, Emily Barnett

**Affiliations:** School of Psychology, Flinders University, GPO Box 2100, Adelaide, SA 5001 USA

**Keywords:** Autism Spectrum Disorder, Theory of Mind assessment, Adults

## Abstract

Deficits in Theory of Mind (ToM)—the ability to interpret others’ beliefs, intentions and emotions—undermine the ability of individuals with Autism Spectrum Disorder (ASD) to interact in socially normative ways. This study provides psychometric data for the Adult-Theory of Mind (A-ToM) measure using video-scenarios based in part on Happé’s (Instructions for theory of mind story task, 1999) Strange Stories test. The final items discriminated IQ-matched adults with ASD from controls on the social but not the physical items. Additional validity data included a two-component principal components solution, correlations with existing ToM scales, and the absence of correlations with self-report measures of empathy and social anxiety (not requiring inferences about the intent of others). The expected group differences in ToM were accompanied by marked variability in the ASD sample.

**Electronic supplementary material:**

The online version of this article (doi:10.1007/s10803-017-3080-x) contains supplementary material, which is available to authorized users.

## Introduction

One of the defining features of Autism Spectrum Disorder (ASD) is some level of impairment in social interaction. This impairment may be the result of difficulties in interpreting or ‘reading’ the verbal and non-verbal social communications of other individuals or in communicating with others in ways that accord with normative expectations (APA 2013). These impairments have often been attributed to a core deficit in Theory of Mind (ToM), a deficit which is reflected in a diminished ability to take the perspective of others and considered by some to be independent of intellectual level (e.g., Baron-Cohen 1995, 2001; Baron-Cohen et al. 1985). It has been argued that a diminished ability to interpret the beliefs, intentions and emotions of others will undermine the individual’s ability to interact in ways that are generally considered appropriate and adaptive for a particular social context. Not all research argues for a ToM deficit as the key mechanism underlying the social interaction impairments seen in ASD (see, for example, Stone and Gerrans 2006; and Van de Cruys et al. 2014). Also, numerous quite specific mechanisms have been examined in an attempt to understand the nature and development of these social impairments, with deficits in areas such as facial and vocal emotion processing, attention, or perhaps simply disinclination rather than a deficit, just a few of the mechanisms considered (e.g., Globerson et al. 2015; Koldewyn et al. 2013; Kuchinke et al. 2011; Nuske et al. 2013; Townsend et al. 1996; Weigelt et al. 2012). To what extent such deficits are manifestations of a ToM deficit or possibly represent core deficits in their own right are perspectives on which researchers likely differ. Disentangling the relative contributions of such deficits from those of some more general social-cognitive deficit(s) to the way in which an individual interprets and responds to social communications from another person is obviously extremely difficult given the diverse array of likely co-varying social-communicative cues emitted in any social exchange. Although various perspectives have been advanced about the mechanisms underlying the social-communicative deficits observed in association with ASD, there has been an enduring focus on trying to assess the extent and nature of ToM deficiencies. We extended prior work by focusing on the development and psychometric evaluation of an adaptation (and extension) for adults with ASD of what has perhaps been the most widely used ToM measure in research into ASD, the Strange Stories test (Happé 1994, 1999; Fletcher et al. 1995). By evaluating the instrument using a large sample of adults with ASD and an IQ-matched typically developing sample we highlighted the extent and variability of ToM deficits in adults with ASD.

## Measuring ToM in Adults with ASD

Numerous instruments have been developed to measure ToM, or some aspect(s) of ToM, in individuals with ASD, although there has been no universally accepted operationalization of ToM. Early research in this area, shaped by research examining ToM in young typically developing children, used variants of the false belief tasks used widely in mainstream developmental research (e.g., Baillargeon et al. 2010; Baron-Cohen et al. 1985; Bowler 1992; Wimmer and Perner 1983). Following the observation that many older children and adolescents with ASD could pass such tests^1^–despite their pronounced social impairments being suggestive of ToM deficits—researchers developed what were considered to be more age-appropriate tests that would be sensitive to those social-cognitive deficits that characterize older individuals. For example, The Reading the Mind in the Eyes Test probes the person’s ability to associate a specific mental state descriptor (e.g., flirtatious, hostile) with the expression conveyed by an image of a pair of eyes (Baron-Cohen et al. 2001). Another test–the Strange Stories (Happé 1994)–comprises a number of scenarios or stories, presented on pencil-and-paper, in which the examinee is required to explain the meaning of the behavior of the key characters within the scenarios when they use expressions that mean something other than what a literal interpretation of the expression might suggest (e.g., metaphors, sarcasm, white lies). Happé’s original instrument comprised 24 mental or social stories (i.e., stories requiring a reading of the social intent of the characters) and 6 control stories (i.e., stories not requiring any social inferences). When compared with IQ-matched controls, individuals with ASD were expected to perform worse on the mental or social, but not the control (or what are referred to as the physical) stories. Sub-sets of items from the Strange Stories test (Fletcher et al. 1995; Happé 1994, 1999) have provided the stimuli for many of the other examinations of ToM deficits in both children and adults with ASD.

Various perspectives appear to have motivated the design of instruments or tests that have been used with adult samples. One perspective has been that ToM deficits in adults may only become apparent when the context mirrors the demands of real life social exchanges. Ponnet et al. (2008) found that ASD-linked deficits in interpreting emotional expressions in actors’ conversations were more pronounced when the context was naturalistic and unstructured rather than highly organized or predictable. This finding aligns with observations that differences in social attention between ASD and typically developing samples only emerged with naturalistic (rather than static) stimuli capturing the dynamics of social interactions (Chevallier et al. 2015). In a similar vein, Frith (2004) suggested that much of the apparent variability in ToM test performance that may be seen in adult samples may reflect the extent to which the task allows an individual to hack out a solution to a problem. Thus, while a person with impaired ToM may be able to work out answers when confronted with pencil-and-paper scenarios, they may experience much greater difficulty when confronted with the limited time constraints that typify an ongoing or live social interaction.

Regardless of the instrument used, however, variability in the ToM performance of individuals with ASD has been suggested, though there is no robust empirical evidence that provides a clear indication as to the extent of such variability in adults. There are no data available to show, at the group level, how adults with ASD compare with IQ-matched non-ASD controls, whether some proportion of individuals with ASD match the ceiling performance of non-ASD controls, what the extent of any overlap between ASD and non-ASD samples may be, and so on. Put simply, there is a paucity of empirical data on the extent and variability of ToM deficits in adults with ASD.

Moreover, there are major limitations with existing ToM measurement instruments, limitations which not only provide major challenges for assessing ToM in adults with ASD but also constrain our understanding of the extent of ToM deficits in adults. A key limitation is that the administration of the instruments has not been standardized and a number of variants of the tests have been used. In the absence of a standardized tool used with large sample sizes, a close psychometric evaluation that would normally be associated with the development of an assessment tool is not possible. There are, of course, a couple of very obvious reasons for why this has occurred. First, many of the ToM instruments used in research with participants with ASD were developed to explore hypotheses about the nature of deficits in ASD rather than to provide a formal assessment device akin to an intelligence or personality test. Consequently, a probing psychometric analysis was not on the test developers’ agendas. Second, researchers generally find it extremely difficult to access the number of individuals with ASD that would make such a psychometric exercise viable. Consequently, reliable normative data are non-existent. These gaps in the existing literature motivated our adaptation, extension and evaluation of the Strange Stories test (Happé 1994, 1999).

## The A-ToM

The current study was designed to address these limitations using the A-ToM (Adult-Theory of Mind), which is an adaptation and extension of the Strange Stories test. In the following section we outline the steps taken in this study to extend the pioneering work involving the Strange Stories in order to provide a robust ToM assessment instrument for adults with ASD and provide an indication of the extent and variability of ToM deficits in adults with ASD. There were several main objectives underpinning the development and evaluation of the instrument. The first objective was to develop a set of test items, each of which involved participants responding to a brief video depicting actors engaged in a naturalistic interpersonal interaction. To simulate the demands imposed by many day-to-day social interactions, participants had to respond to the question posed about each item within 1 min, a constraint which limited their opportunity to hack out a solution to the items (as may happen when there is an opportunity to re-study a pencil-and-paper scenario). A tool that does not provide an opportunity for a person to routinely follow rules or hack out solutions, but requires the monitoring of the unfolding of relationships between characters and the understanding of other subtle social nuances, should provide a more complete understanding of a person’s impairment in this domain.

Our presentation format offers two other potentially important advantages. From a testing and diagnostic perspective, the responses to the video scenarios offer a valuable starting point for discussions between clinicians and clients about their (mis)understanding of social cues and situations, with such scenarios almost certainly having greater face validity from a client’s perspective than pencil-and-paper equivalents. The formal identification of significant deficits in this area may highlight factors that may be constraining the development of effective interpersonal relationships, undermining the individual’s adaptation to the demands of their employment situation, or even contributing to risk for naïve involvement in criminal behavior or maladaptive interactions with criminal justice system professionals (cf. Brewer and Young 2015). And, from a research perspective, such scenarios would allow for the recording of measures such as eye movements (cf. Senju et al. 2009) and reaction time that may be informative about underlying psychological processes. Other researchers have examined ToM measures that required participants to make social-cognitive inferences about interactions observed in short video vignettes or longer movies. Two examples of such tests that have been used with individuals with ASD are the Awkward Moments Test (Heavey et al. 2000) and the MASC (Movie for the Assessment of Social Cognition, Dziobek et al. 2006). Although both instruments showed promise in discriminating adults with ASD from control participants, the sample sizes were so small (<20 per group) that it was obviously not possible to conduct a detailed psychometric evaluation of the instruments or to assess the reliability of the its correlations with other relevant measures.

Consistent with the Strange Stories test, some A-ToM items required participants to draw mental, or social, inferences, while others were physical items. The former should differentiate individuals with ASD from typically developing individuals while the latter should not. Thirteen of the total pool of 17 items evaluated were based on the original Happé (1999) Strange Stories items^2^ although, for some items, the content had to be modified to adapt the item from a pencil-and-paper format to video or digital presentation. A further four social items were developed for this study. The eight physical (control) items were based on the items developed by Fletcher et al. (1995) and fully described by Happé (1999). Although all participants provided data for all of these items, our aim was to use the psychometric data to reduce the set of items to a more manageable size (while maintaining sensitivity) which would allow test administration within no more than 30 min. Decisions about appropriate test length and administration duration are obviously somewhat arbitrary. However, our research and clinical experience suggested that use of the instrument in either of those contexts would be more likely if administration could be effected in 20–30 min. Given the stimuli and the maximum permitted response times, the final test would, therefore, contain in the vicinity of 10–15 items.

The second objective was to evaluate the instrument with large samples of (performance) IQ-matched ASD and typically developing adults of average or above-average intelligence (i.e., IQ ≥ 85). A number of published studies involving adults with ASD have used subsets of the 24 items published by Happé (1994), sometimes adapted or translated (e.g., Happé 1994; Happé et al. 1996; Heavey et al. 2000; Jolliffe and Baron-Cohen 1999; Ponnet et al. 2004; Roeyers et al. 2001; White et al. 2009). Sample sizes for ASD participants in these studies ranged from 5 to 24, with the total number of participants with ASD numbering 139. Note, however, that the total number would have been even lower if some samples had not included some individuals with ages less than 16 years (e.g., Happé 1994) or IQs below 85 (e.g., Happé 1994; Heavey et al. 2000; Roeyers et al. 2001). The IQ-matched control samples in the above studies were of similar size to the ASD samples. In order to provide more reliable normative data (although we realized that we would obviously not have sufficiently large samples to be able to produce specific age norms across the adult years), our approach to participant recruitment in this study targeted 120–200 participants with ASD and 75–100 typically developing control participants. Ideally the two samples would be matched on performance IQ. We acknowledge that persons with ASD have unique cognitive profiles and matching with typical controls is difficult; thus, the use of scales such as the Wechsler Scales is preferred as it taps the diversity in abilities (cf. Mottron 2004). Although there is no standard rule for matching, the purpose of the matching should be considered as should the tasks being undertaken. Performance IQ was our preferred match as it is less affected by the verbal demands of the test and thus thought to be a purer reflection of IQ. If our final samples proved to be closely matched on performance IQ, we would be able to control for any verbal IQ differences between the two samples in statistical analyses. We excluded participants with IQ below 85 to facilitate a neater distinction between ToM deficits (despite the matching) and cognitive processing limitations often observed in association with borderline and lower intellectual functioning.

The third objective incorporated two components. One component involved an examination of individual items in order to refine the pool of items based on a combination of item difficulty with item discrimination index and item-total correlation coefficients. The other involved an assessment of inter-rater and test–retest reliability.

The fourth objective was to explore the validity of the A-ToM. First, principal components analyses (PCA) were conducted to explore the components underlying A-ToM performance. Second, we conducted the comparison of performance differences between the ASD and non-ASD samples on the social and physical tests of the A-ToM. Crucially, as Happé and colleagues have argued (e.g., Happé 1994; Fletcher et al. 1995), the ASD sample should perform significantly worse than IQ-matched controls on the social but not the physical items. Third, we examined the relationship between A-ToM performance and performance on two measures that we expected should clearly discriminate ASD and non-ASD samples, yet did not involve any assessment of ToM abilities. One instrument included three sub-scales from the Interpersonal Reactivity Index (Davis 1983), each of which is a self-report scale tapping a particular aspect of empathy (i.e., perspective taking, empathic concern, and personal distress). The other was the mini-SPIN, a measure used to screen for generalized social anxiety disorder (Connor et al. 2001). Fourth, we examined convergent validity of the A-ToM via the correlation of test performance with two other published ToM measures, the Strange Stories and the Frith- Happé animations (White et al. 2011).

As noted earlier in this paper there has been limited research into ToM deficits in adults with ASD, with the size of the samples making it extremely difficult to evaluate the likely extent and variability of ToM deficits in adults. The net effect of realizing the four objectives for the assessment instrument described above was to provide an indication–based on a much larger sample of individuals with ASD than has been reported in previous research–of the extent and variability of ToM deficits in adults with ASD when compared with a typically developing sample.

## Method

### Participants

The ASD sample comprised 163 individuals (50 female) with a diagnosis of Asperger syndrome (AS) or ASD. Their ages ranged from 16 to 62 years (*M* = 27.0 years, SD = 11.8 years). Scores on the Perceptual Reasoning Index (PRI) and the Verbal Comprehension Index (VCI) of the Wechsler Abbreviated Scale of Intelligence-Second Edition (WASI-II; Wechsler 2011) ranged from 86 to 147 (*M* = 108.7, *SD* = 13.5, 95% *CI* [106.6, 110.7]) and 64–149 (*M* = 103.0, *SD* = 15.0, 95% *CI* [100.7, 105.3], respectively.

All but 12 participants were registered with the local agency that coordinates statewide assessment and provision of services. All individuals registered with the local agency had been diagnosed by two qualified diagnosticians and met DSM-IV-TR [APA 2000] criteria. The other 12 participants were diagnosed either by a psychologist recognized by the local agency or by two qualified diagnosticians. The individuals also met clinical cut-off scores for AS using either the Autism Diagnostic Interview—Revised (Lord et al. 1994), Gillberg and Gillberg’s criteria (Gillberg and Gillberg 1989), or the Childhood Asperger Syndrome Test (Scott et al. 2002). Participants were recruited in three main ways: (1) They had participated in previous psychological studies and volunteered to consider participating in future studies. (2) They responded to a flyer at a local clinical practice specializing in ASD. (3) They responded to a flyer circulated by the local autism agency (*N* = 150). The flyer described the study and its requirements and how to make contact with the researchers. Participants were paid $120 for their participation and were tested on-campus, in their own home or in a local community facility such as a library. Data collection spanned a 34-month period with the objective being to gather as large a sample as possible, but hopefully between 120 and 200 adults to provide an adequately powered set of group contrasts. An additional 32 individuals completed all assessments but were not included in this study because they recorded a PRI on the WASI-II of less than 85. Given (a) the costs associated with the lengthy testing sessions and (b) our pilot testing of the A-ToM in class practical sessions indicated that a smaller sample of non-ASD individuals should deliver stable data, we only targeted 75–100 non-ASD participants. The final sample included 80 typically developing individuals (56 female) who had added their contact details to a register of individuals registered for participation in psychology studies, or elected to participate for course credit. Their ages ranged from 17 to 59 years (*M* = 26.1 years, SD = 10.2 years). Scores on the PRI and the VCI ranged from 86 to 136 (*M* = 106.4, *SD* = 12.1, 95% *CI* [103.7, 109.0]) and 82–160 (*M* = 111.2, *SD* = 13.9, 95% *CI* [108.2, 114.3], respectively. Most were undergraduate students or were enrolled in programs designed to facilitate transition to university study for mature-aged students. To increase the likelihood that we might achieve an approximate IQ match for the ASD and non-ASD samples, we tried to avoid recruiting potential participants who were enrolled in elite undergraduate programs or at advanced stages of study. A further 20 potential control participants were tested but excluded because their AQ-10 score (Allison et al. 2012) exceeded 6 (n = 7), their WASI-II Perceptual Reasoning Index (PRI), was below 85 (n = 4), they had an immediate family member with a diagnosis of ASD (n = 5), or English was their second language (n = 4).

The two groups proved to be nicely matched on the PRI, *t* (241) = 1.28, *p* = 20, *d* = 0.18, 95% *CI* [-0.09, 0.44], but the ASD group was significantly lower on the VCI, *t* (241) = 4.15, *p* < .001, *d* = 0.57, 95% *CI* [0.29, 0.84]. Accordingly, the VCI was used as a covariate in analyses.

### Materials

*A-ToM*. There were two separate sub-tests of items: one comprised 17 social items and the other included 8 physical items. The social items included 13 items based on items from Happé (1994, 1999) and 4 items developed for this study. These 4 items were developed by the second author and stimulated by observations of apparent sources of difficulties experienced and articulated by clients encountered in day-to-day clinical practice. The items evolved from discussions with clients who had shared their social mishaps and *faux pas* in a group setting. Supplementary Materials Table 1 shows the script for all social and physical items. Items 2, 3, 6 and 10 are the new items and they include two *faux pas* and two sarcasm items. The other items were adapted from Happé’s items and included items from categories such as lie, white lie, misunderstanding, double bluff, irony, figure of speech, joke, pretend and persuasion. The 8 physical items were based on those used by Fletcher et al. (1995) and described in Happé (1999). A script was written for each item and the items were then acted out and professionally filmed in order to produce a high quality set of digital stimuli. The scenarios ranged in duration from 14 to 108 s. The six social and six physical items^3^ that comprised the final scale (after the psychometric analyses) can be accessed via the link below:

**Table 1:** Proportion correct (P_C_), item discrimination index (D), and item-total correlation coefficients for ASD and non-ASD control samples on A-ToM social and physical items

Social items	ASD			Non-ASD		
	P_C_	D	Item-total	P_C_	D	Item-total
Bunnies*	0.50	70.7	0.47	0.62	62.1	0.12
**Party**	0.77	48.2	0.51	0.93	30.8	0.54
**Crying man**	0.74	69.0	0.58	0.87	34.8	0.43
**Burglar**	0.24	47.4	0.45	0.40	32.5	0.19
**Hat**	0.76	40.6	0.36	0.93	23.1	0.46
**Spaghetti**	0.63	60.9	0.38	0.83	50.1	0.41
Chocolate	0.57	63.3	0.38	0.75	38.8	0.05
Xmas present	0.65	45.8	0.25	0.85	0.6	−0.02
Dinner	0.68	76.7	0.62	0.77	23.7	0.02
Pregnancy	0.87	28.0	0.35	0.97	15.4	0.35
Walking home	0.42	38.3	0.26	0.47	39.9	0.24
Sausages	0.25	50.8	0.38	0.31	55.6	0.37
Alibi	0.22	50.0	0.27	0.17	21.9	0.06
Cough	0.71	63.9	0.37	0.77	31.4	0.27
Dog	0.26	28.9	0.26	0.22	21.9	0.02
Banana	0.50	58.4	0.31	0.50	55.0	0.17
Vase	0.90	28.2	0.32	0.92	30.8	0.32
Physical Items						
**Light bulbs**	0.59	48.2	0.25	0.70	42.0	0.20
**Swimming**	0.46	85.5	0.32	0.51	75.7	0.29
**Glasses**	0.61	65.4	0.31	0.71	54.3	0.24
**Car**	0.35	67.6	0.33	0.61	47.0	0.28
**Leg injury**	0.82	38.3	0.33	0.76	34.0	0.26
**Librarian**	0.44	73.6	0.34	0.38	51.3	0.10
Burglar alarm	0.92	20.5	0.25	0.91	16.3	0.01
Mayonnaise	0.80	42.9	0.34	0.93	−8.0	−0.20

*Bolded items denote the final pool of items

Physical Playlist Link: https://www.youtube.com/playlist?list=PLJCW1evzKKcuy1rGu3Ocatm97s_KpdhyI.

Social Playlist Link: https://www.youtube.com/playlist?list=PLJCW1evzKKctzHvYfB1RADd27m8IBaWcu.

The 25 scenarios were incorporated into a VLC Media Player playlist in a random order. Four different randomly-ordered versions were created for counterbalancing purposes, with social and physical videos distributed randomly throughout each version. The questions relating to each scenario were displayed on screen following the video, and participants were provided with a response sheet to write down their responses. For questions that had two components (i.e., Is this true? Why did she say this?), participants were provided with a ‘Yes/No’ response in addition to the blank writing space that was provided for all other questions. Participants were instructed at the beginning of the task that they would have 60 s in which to record their response for each question. The test administrator started a timer when the question appeared on the screen and, if the timer went off before the participant had finished answering, they were instructed to stop writing. Selection of this interval was based on a pilot study in which we group-tested 25 upper-level psychology students, recording the time it took for most of the group to complete their response to each question. Participants’ answers were rated on a 0–2 scale: 0 (incorrect), 1 (partially correct) or 2 (correct), with the scoring criteria providing examples for each answer type provided for each item (see Supplementary Materials Table 2 for scoring criteria).^4^

**Table 2:** Component matrix for PCA of the two components solution of the 12 A-ToM items

Item	Factor pattern coefficients		Communalities
	Factor 1	Factor 2	
Spaghetti	**0.70**	0.01	0.53
Party	**0.69**	**0.40**	0.53
Crying man	**0.67**	0.38	0.49
Burglar (glove)	**0.65**	0.21	0.42
Hat	**0.62**	0.15	0.39
Bunnies	**0.36**	**0.53**	0.33
Swimming	0.03	**0.59**	0.36
Lost glasses	0.17	**0.56**	0.31
Car	0.31	**0.54**	0.32
Light bulbs	0.01	**0.54**	0.31
Leg	0.20	**0.51**	0.26
Librarian	0.21	**0.41**	0.18

Major loadings above 0.32 are bolded

#### IQ Control

Participants were administered the four subtests of the WASI-II: Block Design, Vocabulary, Matrix Reasoning, and Similarities. The Block Design and Matrix Reasoning subtests make up the Perceptual Reasoning component, while the Vocabulary and Similarities subtests make up the Verbal Comprehension component. Composite scores are calculated from these to create a Perceptual Reasoning Index (PRI), a Verbal Comprehension Index (VCI), and a Full Scale Intelligence Quotient. Reliability and validity data are reported in McCrimmon & Smith (2012).

#### Discriminant Validity Measures

The Interpersonal Reactivity Index (see Davis 1983, for reliability and construct validity data) comprises four seven-item, self-report sub-scales which tap four different aspects of empathy. We used three of the four scales which measured the extent to which individuals self-reported (a) taking the psychological perspective of others (i.e., perspective taking), (b) showing concern for others in difficulty (i.e., empathic concern), and (c) feeling disquiet about tense interactions with others (i.e., personal distress). Participants read a series of statements and rated to what extent they believed the statement described them (0 = *does not describe me well*; 4 = *describes me very well*), and received a score out of 28 for each subscale.

The Mini-SPIN–based on an item analysis of the Social Phobia Inventory (see Connor et al. 2001, for psychometric data)–is a three item, self-report screener for generalized social anxiety disorder. Participants read three statements and were asked to rate to what extent the statements applied to them (0 = *not at all*; 4 = *extremely*), thus receiving a score from 0 to 12. Although the items on both the Interpersonal Reactivity Index and the Mini-Spin (e.g., “I sometimes try to understand my friends better by imagining how things look from their perspective” or “I often have tender, concerned feelings for people less fortunate than me,” Davis 1983, or “Fear of embarrassment causes me to avoid doing things or speaking to people; ”Connor et al. 2001) seem likely to distinguish ASD and non-ASD samples, they do not appear to involve the type of social-cognitive reasoning associated with reading the subtleties of an unfolding social interaction.

#### Convergent Validity Measures

The two measures used were the Strange Stories test and the Frith-Happé animations (White et al. 2011). Many adaptations of the Strange Stories test have been reported in the literature. We used the eight social and eight physical stories listed in Happé (1999) and used by Fletcher et al. (1995). Two versions, with either the eight physical stories first or the eight social stories first, were alternated across participants. Participants read the story and then answered the appropriate question; there was no time limit within which participants had to respond. Participants’ answers were rated on a 0–2 scale: 0 (incorrect), 1 (partially correct) or 2 (correct).

The other convergent validity measure was a (slightly) modified version of White et al.’s (2011) task which used what are commonly referred to as Frith and Happé’s (Abell et al. 2000) animations. The 14 videos from the original study were put together in a NeuroBehavioural Systems Presentation file: 2 practice videos, 4 ToM (i.e., social or mental) videos, 4 goal-directed (physical) videos, and 4 random videos, and were played in a random order for each participant. Following White et al. (2011), after seeing each video participants viewed a screen that asked them to categorize the behavior displayed by the triangles in the video as indicating either a mental interaction, physical interaction, or no interaction by selecting the corresponding numbers on the keyboard. Participants first completed two practice trials, for which they received feedback on their responses, and were able to read the definitions of the interaction types while they were answering. No feedback was provided on further responses. For mental interaction videos, participants were also asked to select from a list of words the word that best described how each of the triangles were feeling at the end of the video (one question about the feelings of the large triangle, one question about the feelings of the small triangle, with responses giving rise to a feelings categorization score). These additional questions only appeared if the participant had correctly categorized the video as mental; if they were incorrect, they did not receive these questions. Participants received 1 point for correct answers. A score out of 4 was received for correct categorization of each of the 3 types of videos (mental, physical and random); a score out of 8 was received for correctly identifying the feelings of the triangles in the mental videos; and a total score of 12 was possible for correct categorization of all video types. White et al. (2011) found that adults with ASD were less effective than age and IQ-matched controls at identifying mental state interactions and categorizing emotions.

#### Other Measures

Participants in the ASD sample also completed several other measures: the DASS-21 (Lovibond and Lovibond 1995), the AQ50 (ASD participants; Baron-Cohen et al. 2001), and a questionnaire probing the nature and intensity of any restricted interests or preoccupations. These measures were used for screening purposes for a completely different research project and were interposed as fillers to maximize the interval between the A-ToM and the Strange Stories. The non-ASD participants completed the AQ-10 (Allison et al. 2012).

### Procedure

Details of the study were explained to participants and they read a letter of introduction and gave consent. They first provided details about their age, gender, language, ASD diagnosis, and family ASD diagnosis. Tests were administered in the following order: A-ToM (or Strange Stories), Frith-Happé animations, WASI-II, Mini-SPIN, Interpersonal Reactivity Index, DASS-21, AQ50, restricted interests questionnaire, and Strange Stories (or A-ToM). The presentation order of the A-ToM and Strange Stories was counterbalanced.

Depending on the individual participant’s access to transport, testing took place at the university, in a local community library or hall, or in the participant’s home. Testing sites were all within a radius of 120 km of the university. For off-campus testing, the test administrator was accompanied by an upper-level psychology student. There were two test administrators, both of whom had completed honors level studies in psychology. Participants were told they could take breaks when needed during the session. Session durations ranged from 2.5 to 4 h.

#### Inter-Rater Reliability and Test–Retest Stability

To assess inter-rater reliability for the A-ToM and the Strange Stories, two observers (blind to participant’s group) studied, and practiced with, the scoring protocols and, after training, scored 30% (*N* = 73) of the response sheets. To examine stability of the A-ToM, 40 individuals were retested by one of the two administrators at intervals ranging from 2 to 83 weeks (*M* = 23.7 weeks, *SD* = 23.4 weeks).

## Results

### Item Analysis

Our first objective was to conduct an item analysis with a view to reducing the pool of items to a discriminating subset that would be more manageable from a test administration perspective yet still provide adequate coverage of the content domains (e.g., 10–15 items). Table 1 shows the item difficulty (i.e., the proportion of participants in each group who provided the correct response for each item), the item discrimination index (i.e., the proportion of participants in the highest-scoring 25% of each group who answered the item correctly minus the proportion of participants in the lowest-scoring 25% of each group who answered the item correctly), and the item-total correlation coefficient for each of the 25 social and physical items (which provides additional information about the variability explained in test scores).

We first identified social items on which the ASD sample were obviously inferior to the controls. Using a criterion of proportion correct for the ASD sample being more than 0.10 below that for the non-ASD sample, we reduced the pool of social items to eight. Although item difficulty might desirably cluster around 0.5 it was clear that there were (a) few items for which this held for the ASD sample and (b) a number of items on which (quite predictably) the non-ASD sample was close to ceiling. For these eight remaining items our criteria were (a) the proportion correct for the ASD sample should lie between 0.25 and 0.75 (or very close to those boundaries if it allowed us to retain an item), and (b) the item-total correlation for the ASD sample should exceed 0.20 (range = 0.25–0.58), and the item should be clearly more difficult (i.e., the item discrimination index) for the low-scoring than the high-scoring examinees. Given that for two of these eight items, the item-total correlation for non-ASD participants did not even reach 0.10, we reduced the item pool for social items to six, with these being the items bolded in Table 1. The final six items included examples of *faux pas* (1), sarcasm (2), white lie (1), bluff or persuasion (1) and perspective taking or misunderstanding (1). Three of these six items were based on, or adapted from, items described in Happé (1999), and the other three items were from the pool developed for this study.

To produce an equivalent number of physical items, we simply excluded the two items for which the item-total coefficients failed to reach 0.10. For the ASD-sample, this meant that proportion correct for one of these items clearly exceeded our desired cut-off of 0.75. All physical items were based on, or adapted from, items described in Happé (1999). Importantly, the outcome of the reduction of the item pool was such that the social items did not emerge as significantly more difficult overall than the physical items (contrary to what Fletcher et al. 1995, had found for the original Strange Stories physical items) for either the ASD sample (*M* = 0.61, *SD* = 0.21 vs. *M* = 0.55, *SD* = 0.16), *t* (10) = 0.57, *p* = .58, or the non-ASD controls (*M* = 0.76, *SD* = 0.21 vs. *M* = 0.61, *SD* = 0.14), *t* (10) = 1.45, *p* = .18.

### Reliability

Inter-rater reliability, using Cohen’s kappa, was 0.77 for the 6-item A-ToM social scale and 0.88 for the 6-item A-ToM physical scale. The corresponding values for the Strange Stories’ scales were 0.80 and 0.81. Test retest coefficients (*r*) were 0.82 and 0.64 for the A-ToM social and physical scales, respectively.

### Validity

#### Principal Components Analysis

A principal components analysis (PCA) was conducted on the 12 A-ToM items (6 social, 6 physical) using all 243 participants. A Kaiser–Meyer–Olkin measure (KMO = 0.82) exceeding 0.6 (Tabachnick and Fidell 2001) and Bartlett’s Test of Sphericity, *χ* ^*2*^ (66) = 364.7, *p* < .001, respectively, confirmed the adequacy of the sample size and the correlations between items. Given the likelihood of psychological constructs being strongly correlated, we used an oblique rotation, direct oblimin (delta = 0) following Fabrigar, Wegener, MacCallum, and Strahan (1999). The criteria for determining the retention of components combined the Kaiser criterion (eigenvalues > 1.0), the Cattell scree test (for discontinuities in the plot of the eigenvalues), parallel analysis, simple structure and interpretability. A score above 0.32 on a primary loading of items after rotation was used as the cut-off for inclusion in a component (cf. Tabachnick and Fidell 2001).

The PCA indicated three components explaining 25.4, 11.6 and 8.6% of the variance. To confirm the number of components, a parallel analysis (Zwick and Velicer 1986) was conducted using software from Watkins (2000). Eigenvalues produced from a randomly generated data-set of the same size were compared with those from the PCA; if the values for the latter exceeded those from the former those components were retained for subsequent examination. The outcomes of this analysis indicated two components and the PCA was repeated to extract a two component solution. (See Table 2 for complete details of loadings and communalities, and Fig. 1 Supplementary Materials for the scree plot.) Component 1 corresponded to a social component with all six of the purported social items loading on this component, with loadings ranging from 0.70 to 0.36. Component 2 corresponded to a physical component. All six of the purported physical items loaded on this component, with loadings ranging from 0.59 to 0.41, as did two items (party and bunnies) with loadings of 0.40 and 0.53, which also loaded on the social component, with loadings of 0.69 and 0.36. Although these two items were not the most difficult, subjectively they appeared more complex in the sense that they were of longer duration and involved a greater number of conversational transitions between characters than did other items. Corrected item-total correlations for Components 1 and 2 ranged from 0.32 to 0.55 and 0.24 to 0.40, respectively^5^. While the loading of two items on both components indicates that the social and cognitive items did not separate neatly into two completely independent components, the PCA provides a basis for concluding that the two sub-tests were tapping these two different aspects of functioning.

#### Discrimination Between Groups

Table 3 shows the means, 95% CIs, effect sizes and 95% CIs around those effect sizes for the various ToM sub-tests or scales administered to the two groups: that is, the 6-item A-ToM social and physical sub-tests, the Strange Stories social and physical scales, and the Frith- Happé animations.

**Table 3:** Descriptive statistics for the various ToM scales for the ASD and non-ASD control sample

Scale	Group	
	ASD (*N* = 163)	Non-ASD (*N* = 80)
A-ToM: Social		
Mean (& SD)	9.1 (2.4)	10.4 (1.5)
95% CIs	[8.7, 9.4]	[10.1, 10.8]
Cohen’s *d (& 95% CIs)*	0.64 [0.37, 0.92]	
A-ToM: Physical		
Mean (& SD)	7.7 (2.7)	8.2 (2.5)
95% CIs	[7.3, 8.1]	[7.7, 8.8]
Cohen’s *d (& 95% CIs)*	0.22 [−0.05, 0.48]	
Strange Stories: Social		
Social		
Mean (& SD)	12.1 (3.0)	14.1 (2.1)
95% CIs	[11.6, 12.5]	[13.7, 14.6]
Cohen’s *d (& 95% CIs)*	0.76 [0.48, 1.04]	
Strange Stories: Physical		
Mean (& SD)	12.1 (3.0)	14.1 (2.1)
95% CIs	[11.7, 12.6]	[13.2, 14.1]
Cohen’s *d (& 95% CIs)*	0.54 [0.27, 0.81]	
Smith-Happé animations		
Random		
Mean (& SD)	3.4 (0.85)	3.5 (0.67)
95% CIs	[3.3, 3.6]	[3.4, 3.7]
Cohen’s *d (& 95% CIs)*	0.13 [−0.14, 0.39]	
Goal directed		
Mean (& SD)	2.5 (1.0)	2.5 (1.1)
95% CIs	[2.3, 2.6]	[2.3, 2.7]
Cohen’s *d (& 95% CIs)*	0.06 [−0.21, 0.33]	
Mental		
Mean (& SD)	2.7 (1.2)	3.1 (1.0)
95% CIs	[2.5, 2.8]	[3.0, 3.3]
Cohen’s *d (& 95% CIs)*	0.39 [0.13, 0.67]	
Feelings Categorization		
Mean (& SD)	3.7 (2.2)	4.5 (1.9)
95% CIs	[3.4, 4.0]	[4.1, 4.9]
Cohen’s *d (& 95% CIs)*	0.39 [0.13, 0.67]	

The maximum possible scores were 12 (for A-ToM social and physical), 16 (for Strange stories social and physical), 4 (for Frith-Happé animation categorization as random, goal directed and mental), and 8 (for Frith-Happé animation feelings categorization)

The CI and effect size statistics shown in Table 3 suggest that the ASD sample performed worse than the controls on the A-ToM social sub-test, with the group difference effect size much lower for the A-ToM physical sub-test. Given the (non-normal) distributional characteristics of the A-ToM social and physical scores (see Table 4), a binary logistic regression analysis examined whether, after controlling for VCI at Step 1, the A-ToM social score predicted group membership (i.e., ASD vs. control). A series of identical analyses were also conducted with A-ToM physical, Strange Stories Social and Strange Stories Physical individually substituted for A-ToM social. Table 5 summarizes the outcomes of these analyses (B, standard error of B, degrees of freedom, *p* value, Odds ratio or Exp (B) and Nagelkerke R^2^.

**Table 4:** Logistic regression summary statistics for prediction of group membership by VCI (Step 1) and either A-ToM social, A-ToM physical, Strange Stories social or Strange Stories physical (Step 2)

Statistic							
Analysis & Predictor variable	*B*	*SE*	*df*	*p*	*Exp (B)*	*Exp (B) 95% CIs*	*Nagelkerke R* ^*2*^
VCI	0.027	0.011	1	0.012	1.028	[1.006, 1.050]	0.092
A-ToM social	0.325	0.095	1	0.001	1.384	[1.149, 1.665]	0.169
VCI	0.037	0.010	1	0.000	1.038	[1.017, 1.059]	0.092
A-ToM physical	0.041	0.056	1	0.471	1.04	[0.933, 1.163]	0.095
VCI	0.019	0.011	1	0.089	1.019	[0.007, 1.042]	0.092
Strange Stories social	0.381	0.092	1	0.000	1.463	[1.223, 1.751]	0.214
VCI	0.025	0.011	1	0.026	1.025	[1.003, 1.048]	0.087
Strange Stories physical	0.161	0.068	1	0.018	1.175	[1.028, 1.343]	0.120

**Table 5:** Number of participants (and cumulative proportion) obtaining each score on the A-ToM social and physical scales for the ASD and non-ASD control sample

Scale & Group	Score												
Social	0	1	2	3	4	5	6	7	8	9	10	11	12
ASD													
Frequency	0	1	2	4	3	4	10	13	18	17	36	40	15
Proportion^Cum^	0.00	0.01	0.02	0.04	0.06	0.09	0.15	0.23	0.34	0.44	0.66	0.91	1
Non-ASD													
Frequency	0	0	0	0	2	0	0	0	3	9	22	26	18
Proportion^Cum^	0.00	0.00	0.00	0.00	0.03	0.03	0.03	0.03	0.06	0.18	0.45	0.78	1
Physical													
ASD													
Frequency	0	1	4	6	11	16	15	18	28	22	15	12	10
Proportion^Cum^	0.00	0.01	0.03	0.07	0.13	0.23	0.33	0.44	0.61	0.74	0.83	0.91	1
Non-ASD													
Frequency	1	0	0	2	2	5	12	6	10	13	15	9	5
Proportion^Cum^	0.01	0.01	0.01	0.04	0.06	0.13	0.28	0.35	0.48	0.64	0.83	0.94	1

After controlling for VCI, the A-ToM social score significantly predicted group membership, with each one-unit increase on the A-ToM social scale increasing the odds of being classified as a control group member by a factor of 1.38. The corresponding analysis using the A-ToM physical score showed no significant prediction of group membership beyond that provide by VCI. In sum, although verbal IQ clearly contributed to group differences (see Table 5), the A-ToM social, but not the physical, sub-test discriminated the ASD from the non-ASD sample, thereby meeting one of the expectations for the instrument. Note that WASI-II PRI did not contribute to prediction of group membership, as was expected given the matching of groups on PRI, *B*=-0.020, *SE* = 0.011, *df* = 1, *p* = .087, *Exp (B)* = 0.981, CI [0.959–1.003].

Following a reviewer’s suggestion that the ability to understand more complex language (cf. Happé 1995) – perhaps as indexed by the vocabulary (or similarities) component of the WASI-II – may underpin ToM task performance, we ran two separate logistic regressions using vocabulary and similarities instead of VCI at step 1. In both cases, the results (available from the authors)–in terms of patterns of statistical significance, variance explained and odds ratios–were virtually identical to the results described above for VCI.

Table 5 shows the distributions of scores on the two A-ToM sub-tests for each group, with these data providing the first large-sample indication of the degree of overlap on a ToM measure between ASD and non-ASD adult samples. Although some participants in both groups were close to or at ceiling on the social sub-test, this pattern was less marked for the ASD participants. Moreover, extremely low social scores were more prevalent for ASD participants. In other words, there were clear group differences but there was also marked variability within groups. Again, these patterns were not evident on the A-ToM physical sub-test. Interestingly, the respective correlations between the A-ToM social and physical sub-tests and the AQ50, which we used as a filler task, were −0.004 and −0.045, respectively, for the ASD sample, suggesting that it is unlikely the A-ToM was simply reflecting (self-reported) autism severity.

For the Strange Stories, the CI and effect size patterns for the social sub-test were similar in nature to those for the A-ToM, with the two groups clearly differentiated on the social sub-test. The VCI did not predict group membership but, after controlling for VCI, the Strange Stories social score did, with each increase of one-unit on the social scale increasing the odds of being classified as a control group member by a factor of 1.46. In contrast with the A-ToM, the corresponding analysis using the A-ToM physical score did significantly predict group membership after controlling for VCI, with the odds of classification as a control group member rising by a factor of 1.18 with each one-unit physical scale score increase. Thus, for the Strange Stories, even after removing VCI, group membership was differentiated by both the social and physical sub-tests rather than by the social sub-test alone, a finding which undermines the discriminative capacity of the Strange Stories for adult samples.

For the Frith- Happé animations, the other instrument included to examine convergent validity, the effect size indices shown in Table 3 suggest small group differences, in the expected directions, on the mental and feelings categorization (but not the random or goal-directed) scales. However, the effects were relatively weak and one way ANOVAs, with VCI as a covariate, on each of these scales did not reveal significant main effects for group, *F* (1, 236) = 0.01–3.80, *p* = .053-0.94, partial *η* ^*2*^ = 0.00–0.02. Again, however, VCI contributed significantly for each variable, *F* (1, 236) = 5.37–22.42, *p* = .00–-0.02, *η* ^*2*^ = 0.02–0.09.

As well as requiring the two groups to be distinguished on the A-ToM social, but not the physical, sub-test, we predicted that the three self-report scales of empathy from the Interpersonal Reactivity Index (Davis 1983) and the mini-SPIN screener for generalized social anxiety disorder (Connor et al. 2001) would show group differences and yet not be meaningfully related to the A-ToM social scores (i.e., discriminant validity). On the perspective taking, empathic concern and personal distress sub-scales of the Interpersonal Reactivity Index, the ASD sample performed worse^6^: that is, they were less likely to report taking the psychological perspective of others and expressing concern for others in difficulty, and more likely to report being distressed over problematic interactions with others, *t* (235–241) = 2.67–6.94, *p* < .001. They also reported significantly higher levels of social anxiety, *t* (240) = 5.57, *p* < .001. (Supplementary Materials Table 3 shows the means, 95% CIs, effect sizes and CIs around those effect sizes for these various scales for the two groups). Yet, as hypothesized, there were no significant relationships between any of these four measures and the A-ToM social scale, −0.01 < *r* < .13. (The full correlation matrices for ASD, control and combined samples are provided in Supplementary Materials Table 4.) Nor were there any significant relationships between these scales and the other ToM measures used to examine convergent validity (see below).

#### Convergent Validity

Tables 6, 7 and 8 show the inter-correlations between the A-ToM social and physical sub-tests and other ToM measures, namely, the Strange Stories social and physical sub-tests and the Frith- Happé animations mental and feelings categorization scales for the ASD, non-ASD and combined samples, respectively. In the ASD sample the expected positive relationships emerged between the A-ToM social sub-test and the other ToM measures, as did the relationship between the A-ToM and Strange Stories physical sub-tests. For the control sample, the A-ToM social and physical sub-tests correlated significantly with the corresponding Strange Stories sub-tests, though no relationships were detected for the Frith- Happé animations mental and feelings categorizations scales, likely due to range restriction on both variables. Note also that, for the ASD sample, there were significant and meaningful correlations between all ToM sub-tests and the IQ measures. As Tables 6, 7 and 8 shows, the trend was for correlations with VCI to be a little stronger than for PRI, and for the social-VCI correlation to be a little stronger for the Strange Stories than for the A-ToM.

**Table 6:** Inter-correlations of A-ToM scales with other ToM measures and WASI-II verbal and performance IQ for ASD sample

Measure	1.	2.	3.	4.	5.	6.	7.	8.
1. A-ToM social	–	0.42**	0.58**	0.53**	0.22**	0.34**	0.38**	0.12
2. A-ToM physical		–	0.37**	0.61**	0.14	0.29**	0.33**	0.20**
3. SS social			–	0.62**	0.18*	0.27**	0.46**	0.18*
4. SS physical				–	0.16*	0.31**	0.56**	0.25**
5. Frith-Happé mental					–	0.79**	0.21**	0.26**
6. Frith-Happé feeling						–	0.33**	0.20**
7. WASI-II VCI							–	0.39**
8. WASI-II PRI								–

*p < .05, **p < .01 (2-tailed)

**Table 7:** Inter-correlations of A-ToM scales with other ToM measures and WASI-II verbal and performance IQ for non-ASD sample

Measure	1.	2.	3.	4.	5.	6.	7.	8.
1. A-ToM social	–	0.27*	0.50**	0.41**	0.17	0.12	0.20	0.07
2. A-ToM physical		–	0.24*	0.42**	0.10	0.08	−0.07	0.07
3. SS social			–	0.58*	0.04	0.11	0.10	0.22
4. SS physical				–	0.09	0.12	0.21	0.17
5. Frith-Happé mental					–	0.80**	0.22	0.20
6. Frith-Happé feeling						–	0.22	0.28*
7. WASI-II VCI							–	0.21
8. WASI-II PRI								–

*p < .05, **p < .01 (2-tailed)

**Table 8:** Inter-correlations of A-ToM scales with other ToM measures and WASI-II verbal and performance IQ for ASD and non-ASD samples combined

Measure	1.	2.	3.	4.	5.	6.	7.	8.
1. A-ToM social	–	0.42**	0.60*	0.54**	0.25**	0.33**	0.39**	0.08
2. A-ToM physical		–	0.35**	0.56**	0.14*	0.24**	0.23**	0.16*
3. SS social			–	0.64**	0.19**	0.28**	0.42**	0.15*
4. SS physical				–	0.18**	0.30**	0.50**	0.20**
5. Frith-Happé mental					–	0.80**	0.25**	0.22**
6. Frith-Happé feeling						–	0.33**	0.20**
7. WASI-II VCI							–	0.30**
8. WASI-II PRI								–

*SS social* Strange Stories social, *SS physical* Strange Stories physical, *Frith-Happé mental* Frith-Happé mental state categorization, *Frith-Happé feeling* Frith-Happé feeling categorization, *WASI-II VCI* WASI-II verbal comprehension index, *WASI-II PRI* WASI-II perceptual reasoning index

*p < .05, **p < .01 (2-tailed)

## Discussion

Building on prior research using the Strange Stories test, our objective was to develop a standardized, reliable and valid ToM measure that could be administered in no more than half an hour, would require participants to interpret interpersonal interactions as they play out and without the benefit of extended reflection, and yet still sample the social and physical content domains adequately. Following a process of item-analysis designed to reduce the original 17-item social item pool to items that (a) were more difficult for ASD than non-ASD individuals (but not at ceiling or floor accuracy levels), and (b) showed strong discrimination of good and poor performers with acceptable correlations with the total score, we identified six social items. These included three based on items contained in previous measures and three new items. The physical item pool was resolved by simply eliminating two items from the eight-item pool that did not correlate with the total score. Given the item selection criteria, the net result was a social sub-test that differentiated ASD and non-ASD participants. In contrast, the physical sub-test met expectations in that it did not differentiate the two groups. Importantly, the differentiation on the social scale was maintained after controlling for verbal IQ, despite clear differences in VCI between the two groups and a significant relationship between VCI and the A-ToM social scale.

The data also provided an indication of the extent and variability of ToM deficits in adults with ASD which to date has been lacking in the absence of a psychometrically robust measure of ToM. Despite the clear group differentiation on the A-ToM social scale, the variability in ToM performance within the ASD sample was substantial. Some individuals clearly had little difficulty interpreting the social interactions accurately; others were quite markedly impaired. Thus, while a ToM deficit may be indicative of ASD in adults, the lack of one (at least as detected on an instrument such as the A-ToM) may not preclude a diagnosis. If indeed this proved to be the case it would call into question whether a ToM deficit should be considered a core feature of the disorder (at least in adulthood). Further, while one may expect this deficit to be related to autism severity, the lack of a relationship with the AQ scale suggests this may not be the case.

The examination of reliability and validity confirmed the potential of the ToM measurement instrument. Inter-rater and test–retest reliability were acceptable, especially given the interval between tests. Principal components analysis confirmed the presence of relatively neat social and physical components. Additionally, with the benefit of a much larger sample size than has previously been used in this area of research, the other measures of ToM–the Strange Stories and the Frith-Happé animations–did not show the same differentiation between ASD and non-ASD adults that was exhibited on the A-ToM. On the Strange Stories, the groups were clearly differentiated on the social scale–but they were also differentiated on the physical scale and the differentiation on both scales persisted after controlling for VCI. Although the ASD group performed worse on the mental and feelings measures of the Frith-Happé animations, the effect sizes were relatively weak and the differences were not significant with VCI controlled. It is important to note, of course, that the Strange Stories and the Frith-Happé animations have not been the beneficiaries of any item analysis approach such as that carried out here with the A-ToM, nor could they have been given the limited sample sizes in their supporting studies. It is conceivable that a similar approach to producing a final item set on those scales could produce instruments that provide the same differentiation, although our data suggest that the number of items preserved in such a revision would likely be quite small.

Other indicators of convergent and discriminant validity were detected. First, there were significant correlations between the A-ToM scales and the corresponding scales from the Strange Stories and the Frith-Happé animations. Second, the expected group differences emerged on the empathy and social anxiety scales of the Interpersonal Reactivity Index and the mini-SPIN, yet these measures were uncorrelated with the A-ToM social scale.

Our examination of the psychometric characteristics of the A-ToM was based on adult sample sizes far larger than used in any previous ToM instrument work in this area. Our data suggest that the instrument has considerable potential for use with ASD adults as a pointer to the types of deficits that may constrain the adaptiveness of their social interactions in a variety of situations. Moreover, the characteristics of the stimuli are such that examinees are likely to perceive the scenarios as realistic (i.e., as having face validity, although we did not formally assess this), thereby providing a plausible starting point for client-clinician discussions about the nuances of social interaction and the interpretation of the interpersonal behaviors of others. And, as we noted earlier, it would be possible for researchers to use the stimuli in conjunction with potential indicators of underlying processes such as response latency or eye tracking.

Despite these positives we believe that it will be crucial for subsequent work to pursue a number of important follow-up questions. First, although our sample of adult ASD participants far outnumbered those reported in the literature to date, it is obviously not large enough to provide normative data partitioned by factors such as age and gender. Second, the scenarios used were all presented as relatively short duration, discrete stimuli. This means, of course, that we are unable to comment on how individuals might respond when they have much more contextual information about the characters they are observing. It is possible that more prolonged exposure to the individuals depicted in scenarios might allow individuals with ASD to learn how to read the behaviors of others. Alternatively, it may simply accentuate the gap between ASD and non-ASD adults. Such information would, of course, indicate if these skills can be learned through extensive exposure and provide potentially important information from an intervention perspective.

Third, the A-ToM is a general measure in the sense that the items require observers to interpret a range of different categories of behaviors (e.g., *faux pas*, sarcasm), with these categories mirroring the focus of earlier work in this area. But, just as our sample showed considerable variability in their global performance, so may it be that adults with ASD have specific deficits (or strengths) in ToM, a possibility canvassed extensively by Brewer and Young (2015). Some individuals may have a particular problem interpreting sarcasm, others may struggle with *faux pas* or metaphors or bluff. And, it will likely be difficult (perhaps impossible) to tease out which of these problems reflect difficulties in decoding the social intent of others vs. some more specific linguistic impairment. In other words, to understand the ToM deficits of individuals with ASD we may well need to undertake much more systematic probing of specific areas of ToM. This is an area that we are actively pursuing via the development of larger item banks that tap into such areas.

Fourth, while our validation efforts included an examination of the structure of the instrument and various aspects of convergent and discriminant validity, a major objective now should be to examine the relationship between A-ToM test performance and completely independent criterion-related measures of social-cognitive functioning. At present, the broader functional significance of the ToM deficits suggested by such tests as the A-ToM and the Strange Stories is assumed, or sometimes inferred from clinical observation, rather than empirically demonstrated. Consequently, the nexus between specific areas or levels of severity of ToM deficit and the individual’s day-today functioning in specific areas, or indeed autism severity, is poorly understood. It is of course possible that future research that proceeds in this direction will reveal that a global measure like the A-ToM adequately predicts the severity of any area of ToM deficit and a broad range of difficulties that an individual with ASD may experience in daily life. But, equally, such research may show an urgent need for much more precise or targeted measures of ToM and for an examination of its impact on adaptive skills.

Finally, there are of course fundamental theoretical issues to be resolved. As we indicated at the outset, various other mechanisms have been posited as crucial for understanding the social interaction impairments seen in ASD (e.g., deficits in processing facial and vocal emotion expressions, pragmatic aspects of language, executive functioning deficits.) Some may prove to be manifestations of a ToM deficit and some may emerge as independent core deficits. A substantial research effort will be required to elucidate the independent and interactive contributions of such factors, with the answers quite likely having implications for the structure of measurement instruments such as that described in this study.

## Electronic supplementary material

Below is the link to the electronic supplementary material.

Supplementary material 1 (DOCX 119 KB)

## Footnotes

1 Note, however, various strands of evidence indicating that explicit prompting may be required for adults to recognize or track the mental states of others rather than it occurring spontaneously (e.g., Schneider et al. 2013; Senju et al. 2009).

2 The Acknowledgments section contains full details on permissions.

3 In the final set of scenarios some actors overlap in some scenes. Whether participants’ overall performance was facilitated (through repetition) or impaired (because those particular actors were ‘harder to read’) by this overlap is impossible to determine.

4 It is possible that group differences in performance were influenced by factors such as the requirement to provide written (rather than verbal) responses or having to draw the appropriate social inferences from short rather than long duration video clips.

5 Separate PCAs run on the six social and six physical items each identified a single component on which all six items’ loadings ranged from 0.51 to 0.74 and 0.43-0.62 for the social and physical sub-tests, respectively.

6 When VCI was entered as a covariate in a between-groups ANOVA, it had no meaningful effect on any of these measured, with all *F* values < 1.
