# Supplementary material for: Measuring Theory of Mind in Adults with Autism Spectrum Disorder
Source: J Autism Dev Disord. 2017 Mar 9;47(7):1927–41. doi: 10.1007/s10803-017-3080-x (PMC5487761; doi:10.1007/s10803-017-3080-x)
Supplement: Supplementary file 1 — Supplementary material 1 (DOCX 119 KB) [file 10803_2017_3080_MOESM1_ESM.docx]

**Supplementary Materials**

Table 1

*Script for A-ToM items, corresponding Strange Stories item, and item type*

*Social Items*

| A-ToM Item | Strange Stories Item / Item Type | A-ToM Script |
| --- | --- | --- |
| 1. Bunnies | Kittens / Persuasion | Two women sit in their living room discussing their bunnies.  SUSIE: “So you know there is a lady coming over today to take a look at the rabbits.”  MRS SMITH: “That’s good, because you know we can’t keep them all.”  SUSIE: “I know.”  She looks sad as she picks up one of the bunnies and cuddles it.  SUSIE: “I just love them so much. I can’t bear the thought of anything bad happening to them. They’re just so beautiful and cuddly.”  A girl approaches the house and knocks on the front door. The door opens to reveal woman 1 and woman 2.  POTENTIAL BUYER: “Hi I’m here to look at the bunnies.”  SUSIE: “Of course, come inside.”  Mrs Smith, Susie and the potential buyer are sitting in the living room. The potential buyer is cuddling one of the bunnies.  POTENTIAL BUYER: “Oh they are all so cute. It’s a shame they’re all have males though, I was really looking for a female bunny.”  SUSIE: “Oh that is a shame. You know if I can’t find a good home for them, I’m going to have to drown them.”  The potential buyer looks shocked and cuddles the bunny closer.  Fade to black  Q: Why does she say she will have to drown the rabbits? |
| 1. Party | Faux Pas | Simon and Dave are standing in the corner of a party.  SIMON: “So my brother knows the guy who owns this place.”  DAVE: “That’s funny, my brother is the guy who owns this place.”  They laugh together.  SIMON: “Nice. I know this might be a bit forward, but I was wondering if I could grab your number?”  DAVE: “Sure, but if you don’t mind, can you not tell anyone about it, as my father doesn’t know I’m gay. Only my brother knows.”  SIMON: “Yeah that’s cool, I know it’s hard. My family knows but they seem pretty chill with it.”  On the other side of the room Rob and Pete, are chatting to Dave’s Dad.  ROB: “So, Mr Jones it looks like my brother and your son are really hitting it off. They make a cute couple.”  PETE (trying to cover it up): “Ah... Rob did you watch that footy game last night?”  Dave’s dad ignores what Pete says.  DAD (To Rob): “Sorry, ‘hitting it off’? What are you implying?”  ROB (Realising what he has said) “Uh, nothing.”  He turns and faces Pete.  ROB: “Yeah, I saw the game! It was epic.”  Fade to black.  Q: Was there anything awkward or uncomfortable in this interaction? If so, what was it? |
| 1. Crying Man | Sarcasm | A man is seen sobbing in on the couch. Sally and Drew are chatting across the room.  DREW: “What’s the matter with him?”  SALLY: “His wife just left him for a younger man.”  DREW: “Oh no, is he doing okay?”  The man bursts into tears dramatically, as Sally and Drew are watching him  SALLY: “Yeah, he’s doing just fine.”  Fade to black.  Q: Is this true? Why did she say this? |
| 1. Burglar (glove) | Burglar / Misunderstanding | A burglar is robbing a suburban house, and is seen taking valuables and money. He climbs out of the window of the house, and runs down the street. He runs past a policeman on his beat, and drops his glove. The policeman sees the burglar drop his glove, and picks it up and begins to run after the burglar.  POLICEMAN: “Stop, you dropped your- -”  Before the policeman can even finish his sentence, the burglar stops running, puts his hands up and interrupts him.  BURGLAR: “Okay. You got me. I broke into the house.”  Fade to black:  Q: Why does the burglar give himself up? |
| 1. Hat | Hat / White Lie | Two girls are sitting at a table drinking coffee.  ANNA: “Have you seen Aunty Jane in that silly hat?”  JOAN: “I thought you loved Aunty Jane.”  ANNA: “I do. It’s just that hat is ridiculous.”  Aunty Jane approaches the girls. She is wearing the hat.  JOAN & ANNA: “Oh hi Aunty Jane!”  AUNTY JANE: “Hi. How do you like my new hat?”  ANNA: “It’s really nice, I love it!”  Fade to black  Q: Why does she say she loves the hat? |
| 1. Spaghetti | Sarcasm | A child and his mother are sitting at a table eating spaghetti. The boy is sitting with a full plate of food in front of him, pushing it around with his fork. His mother stands up after finishing her meal. She goes to take his plate.  MOTHER: “Okay, have you finished that meal there Isaac?”  CHILD: “Yes.”  A close up of the bowl reveals it is full of food.  MOTHER: “Well. That meal must have really filled you up.”  Fade to black.  Q: When the mother said ‘that meal must have really filled you up’, did she mean it? If not, why did she say it? |
| 1. Chocolate | Brothers / Double Bluff | Jim’s dad is sitting at the kitchen table reading the newspaper. Jim storms in.  JIM: “Dad, where are the chocolates?”  DAD: “What chocolates?”  JIM: “Dad, I know you’ve hidden them. Where are they?”  DAD: “Well maybe I hid them because I didn’t want you eating them all.”  JIM: “Please Dad, I just want one. I’ve looked everywhere.”  DAD: “Well, obviously not everywhere.”  Jim: “Okay. I’ve looked everywhere except under the bed and in the cupboard.”  DAD: “They’re under the bed.”  Jim immediately ignores his dad and goes straight to the cupboard. He opens it up and finds the box of chocolates. He takes them, glares at him dad, and then storms out.  Fade to black  Q: Why does he look in the cupboard for the chocolates? |
| 1. Christmas Present | Rabbit / White Lie | It is Christmas morning. Hannah runs into her brothers’ room and jumps on her brother’s bed.  HANNAH: “Isaac! Isaac! Wake up! It’s Christmas, and I’m so excited because I’m going to get a bunny rabbit!”  MUM (calling from offscreen): “Hey kids! Santa’s been, there’s presents!”  The children run out of the bedroom and down the stairs.  The family are sitting around the lounge room. Hannah’s Dad hands Hannah large wrapped box.  DAD: “Here you go Hannah, Happy Christmas.”  Hannah excitedly rips open the wrapping paper and opens the box. Her face drops, and she pulls out a large encyclopaedia.  DAD: “What do you think of your book Hannah?”  HANNAH (trying to put on a happy face): “It’s good Dad, it’s just what I wanted.”  Fade to black.  Q: Is this true? Why does Hannah say the present is just what she always wanted? |
| 1. Dinner | (unnamed) / Irony | Anne sits slumped on the couch with her Dad, watching TV. Anne’s mum is in the kitchen, and brings over their meals.  MUM: (handing the plate of food to the father) “Here you go.”  DAD: “Oh thanks darling, that looks lovely.”  MUM: (handing Anne her plate of food) “And I’ve got your favourite..”  Anne doesn’t acknowledge her mum at all, and continues to stare straight ahead. Her mother waits for a few moments, waiting for Anne to say thank you. Anne says nothing.  MUM: “Well, don’t you just have lovely manners? That’s what I call politeness.”  Fade to black.  Q: Is this true? Why does the mum say this? |
| 1. Pregnancy | Faux Pas | Caroline arrives at her friend’s house and knocks on the front door. Caroline’s friend opens the door.  FRIEND: “Hi Caroline!”  The girls hug.  CAROLINE: “Hi, how are you”  FRIEND: “I’m good thanks, how are you?”  CAROLINE: “Good!”  FRIEND (looking at Caroline’s stomach): “Oh my god, congratulations!”  CAROLINE (looking confused): “Congratulations on what?”  FRIEND: “On your pregnancy..”  CAROLINE: (crossing her arms over her stomach and looking unimpressed) “I’m not pregnant”  FRIEND: “Oh…I’m sorry…come in”  Caroline enters the house.  Fade to black.  Q: Was there anything awkward or uncomfortable in this interaction? If so, what was it? |
| 1. Walking Home | Mrs Peabody / Misunderstanding | Mrs Peabody is saying goodbye to her friend as she is leaving her friend’s house.  FRIEND: “Okay, see you later.”  MRS PEABODY: “Oh, actually, would you mind walking me home? I’m a little bit frightened of getting mugged.”  FRIEND: “I normally would but I’m waiting for the kids to get home and I just don’t have time tonight. I’m sorry.”  MRS PEABODY: “Oh okay.”  FRIEND: “You’re really paranoid aren’t you? You’ll be okay, it’ll probably do you good. Bye.”  MRS PEABODY: “Thanks. Bye.”  The friend shuts the front door and Mrs Peabody leaves.  Later on Mrs Peabody is walking along a dark street alone. A man who is jogging approaches her and begins to speak.  MAN: “Excuse me, do you have the time?”  Without even listening to what the man has said, Mrs Peabody reacts in fright.  MRS PEABODY: “Just take my bag, don’t hurt me!”  She shoves her purse into his hands and hurries off. The man looks very confused.  Fade to black.  Q: Why does she say this to the man? |
| 1. Sausages | Sausages / Persuasion | Two children, Brian and Scott, are lined up in the kitchen of a house, waiting to be served sausages. Brian sees the children in front of him only receive 1 sausage in bread.  BRIAN: “Is that all we get?”  SCOTT: “What do you mean? That’s plenty.”  BRIAN: “Yeah, but I’m starving.”  SCOTT: “You’re always starving. You’re just greedy. You know your mum’s going to get you some food when you get home.”  BRIAN: “Yeah, but sausages are my favourite.”  Brian makes it to the front of the line.  WOMAN (serving sausages): “Here you go, just one.”  BRIAN: “Actually, could I please have two? Because I won’t be getting anything from my mum tonight.”  The mum takes his sausage back to add an extra sausage to his bread.  Fade to black.  Q: Why does the boy say this? |
| 1. Alibi | Armies / Double Bluff | Steve and Pete are sitting at the bar having a drink. Steve finishes off his beer and goes to leave. Pete stays seated.  STEVE: “I’m off mate, are you coming?”  PETE: “Nah that’s alright mate, I might stay and have another drink.”  STEVE: “Alright Pete, I’ll see you at work.”  PETE: “See ya mate.”  Steve heads for the door while Pete remains at the bar.  Steve exits the door of the pub and is approached by a policeman.  POLICEMAN; “Steve, where’s your mate Peter?  STEVE: Why do you ask?”  POLICEMAN: “There’s been a robbery and I think he may have been involved. And I don’t want him to have enough time to get an alibi with his mates.”  STEVE: “Look, I don’t know where he is, and if I did, why would I tell you? He’s my best mate, I hope he has had a chance to talk to the others.”  POLICEMAN: “Listen, don’t make things difficult. He’s either in the pub, or the pool hall, now what is it?”  STEVE: “Fine, he’s at the pub.”  POLICEMAN: “Ahh” (appears to think about Steve’s answer)  Fade to black.  Q: Why does the man tell the policeman that his friend Pete is in the pub? |
| 1. Cough | (unnamed) / Figure of Speech | Brad and his father are sitting in the kitchen Brad starts to cough.  DAD: “Sounds like you’ve got a frog in your throat.”  Fade to black.  Q: Is this true? Why does the father say this? |
| 1. New Dog | (unnamed) / Joke | Hannah and her mum are walking up to the front door of Hannah’s friend James’ house.  MUM: “Are you excited to see James?”  HANNAH: “Yes, he’s got a new dog, and I love dogs!”  Hannah knocks on the door. James opens the door, with a very large dog.  JAMES: “Hi Hannah.”  HANNAH: “Wow that’s not a dog, that’s an elephant!”  Fade to black.  Q: Is this true? Why does Hannah say this? |
| 1. Banana | (unnamed) / Pretend | Katie and Isaac are sitting on the floor of their lounge, playing with toys. Isaac picks up a plastic banana.  ISAAC: “Hey Katie, look. This banana is a telephone.”  Isaac puts the banana up to his ear and pretends to speak into it and press buttons on it.  Fade to black.  Q: Is this true? Why does the boy say this? |
| 1. Vase | (unnamed) / Lie | Hannah is running through her house kicking a soccer ball. She accidentally kicks the ball into a vase, knocking it over and smashing all over the floor.  Hannah’s mum comes through the front door to see the broken vase on the floor. The family dog wanders over.  HANNAH: “It wasn’t me mum. It was the dog!”  Fade to black.  Q: Is this true? Why did she say it was the dog? |

*Physical Items*

| A-ToM Item | Strange Stories Item | A-ToM Script |
| --- | --- | --- |
| 1. Light bulbs | Light bulbs | John is looking at light bulbs, a sales assistant approaches him.  ASSISTANT: “Excuse me sir, can I help you there?”  JOHN: “Yes, I’ve just bought a new for my desk, and I need a new light bulb for it.”  ASSISTANT: “Oh right, okay, well you can buy the Litebrite here, which comes in a single, or you can pay just a little bit more and get the Everbright, which comes in a pack of ten.  JOHN: Well I only need the one, but I think I will take the pack of ten. Thank you.”  ASSISTANT: “Have a good day”  Fade to black.  Q: Why does he buy the pack of 10? |
| 1. Swimming Competition | Armies | A boy and girl sit on the beach looking at the ocean.  HARRY: “Oi, wanna have a swimming race?”  HANNAH: “Uh, yeah, sure.”  HARRY: “I’m definitely going to win.”  HANNAH: “Uh no you’re not, I’m a much better swimmer.”  HARRY: “You’re a better than me in the pool, but I always win in the ocean.”  HANNAH: “Okay then, I’ll race you to the jetty and back.”  HARRY: “Ready, set, go!”  They jump up off the sand and run out into the ocean.  Fade to black.  Both children are running out of the water, with Harry in front. Harry throws his arms into the air.  HARRY: “And Harry wins! See? I told you I could beat you.”  Fade to black.  Q: Why did Harry win? |
| 1. Lost glasses | Glasses | Sarah is looking around for her glasses. Ted is sitting on the couch watching television  SARAH: “Ted, have you seen my reading glasses?”  TED: “When did you last have them?”  SARAH: “I had them yesterday evening when I was looking at the TV programs. Can you help me find them please?”  Ted picks up a piece of paper next to him, looks underneath, sees nothing, then looks straight back at the TV.  TED: “Can’t find them.”  SARAH: “Seriously Ted. I need them.”  Ted gives in, switches off the TV and stands up.  TED: “Fine. Try retracing your steps. What did you do today?”  Sarah thinks for a moment.  SARAH: “Well I went to my early morning fitness class, then the post office, and the flower shop.”  Ted grabs his car keys and heads for the door, without wasting a second.  TED: “Come on then, we’ll try the post office first.”  Fade to black.  Q: Why is the post office the most likely place to look? |
| 1. Car | Car | A man is at a car dealership looking at a new car for sale. He is wearing an expensive suit, suggesting he is well off.  DEALER: “So have you decided?”  MAN: “I’ll take the car, I really like it. I’ve got enough money, so I’ll go down to the bank and get it.”  DEALER: “You can pay the car off over a 12 month period with monthly instalments.”  The man completely disregards the offer, knowing it will just end up costing him more.  MAN: “Oh no that’s fine, I’ll pay in full. I’m sure you guys charge interest.”  DEALER: “Well we do charge interested, but it’s only 5%.”  MAN: “Oh good. I get 8% in the bank, so in that case, I’ll pay in monthly instalments.”  DEALER: “Does this mean you’ll take the car?  MAN: I’ll take the car.”  DEALER: “Congratulations.”  MAN: “Thank you very much.”  DEALER: “It is a very beautiful car.”  The men shake hands.  Fade to black.  Q: Why does he accept the dealer’s offer to pay in monthly instalments? |
| 1. Leg injury | Leg | An older lady steps into the doctor’s office.  DOCTOR “Hi, hello, how are you?”  LADY: “Hi.”  DOCTOR: “Have a seat. What can I do for you today?”  LADY: “Well yesterday I fell over on my icy door step. I did get up straight away, although I did feel quite shaken and bruised. And when I woke up this morning I could scarcely walk. And my leg feels really stiff.”  DOCTOR: “Hmm, let me take a look, and let me know if you feel any pain.”  She analyses the swollen leg, looking quite concerned.  DOCTOR: “It looks quite swollen. I’m going to have to send you to the casualty department at the hospital, and they’re going to need to take an x-ray.”  Fade to black.  Q: Why does she need an x-ray? |
| 1. Librarian | Mrs Simpson | Mrs Simpson, a librarian sits at her desk, using the computer. A boy approaches her holding a book.  BOY: “Hi Mrs. Simpson, I have a book here you may want to put in your library. Which section would you like to put it in?”  MRS SIMSPON: “Well our library has a lot of different sections, what’s the book about?”  BOY: “It’s about plants and their medical uses. It’s heavily illustrated.”  MRS SIMPSON: “Ah I know the perfect place for it.”  BOY: “Are you going to put it with the rest of the books on botany or medicine?”  MRS SIMPSON: “No, I have a special room for this book, where all the books are kept in special cases at a constant temperature. I think I’ll put it in that room.”  Fade to black.  Q: Why Mrs Simpson put the book in a special room? |
| 1. Burglar Alarm | Burglar | A burglar wearing a balaclava approaches the door of a house. The houses lights are off. He approaches the door. There is a sign on the door, which the burglar stops to read it.  It reads: “WARNING! This property is alarmed. Any movement will trigger alarm system!”  The burglar begins to skilfully pick the lock, and enters the house.. He looks up at the motion sensor at the top of the wall. The motion sensor remains off, so far he is undetected. The burglar begins to slowly sneak along the wall, trying not to set off the alarm. He bumps into a table and knocks a vase, which begins to wobble. He catches and steadies it before it falls. He looks up at the motion detector. It is still inactive.  He sighs in relief, and begins to continue creeping through the house. Suddenly he stops - he spots a mouse from across the room. He watches it run along the floor. The motion sensor blinks red. In an instant the alarm begins to blare and lights flash across the room. The burglar quickly runs out the door.  Fade to black.  Q: Why did the alarm go off? |
| 1. Mayonnaise | Mayonnaise | A lady is cracking eggs in the kitchen, separating the yolk from the egg white. Her son stands next to her observing.  MOTHER: “Okay, so to make mayonnaise, what we need to do is separate the whites from the yolks, because to make mayonnaise we only need the yolks.”  SON: “So are we just going to waste all the whites?”  The mother thinks for a moment, until she strikes an idea.  MOTHER: “Um, actually, why don’t you look up recipe for meringues?”  The boy nods, and begins to flip through the recipe book.  Fade to black.  Q: Why does she suggest to make meringues? |

Table 2

*A-ToM Scoring Criteria*

*Social items*

*Bunnies “Why does she say she will have to drown the rabbits?”*

2= reference to persuasion, manipulating feelings, and MUST include reference to trying to induce pity/guilt/make her feel bad, encourage to buy etc.

e.g., ‘To persuade/convince the girl to take the rabbit’, ‘To make the girl feel guilty and take a rabbit’, ‘She is lying to try to guilt her into taking a rabbit’, ‘Trying to pressure her’.

1= reference to outcome (to sell them), or simple motivation (to make Jane sad). Or make clear statement wasn’t true.

e.g., ‘To get the girl to buy/take one’, ‘To get rid of them’, ‘She couldn’t keep them’.

0 = reference to general knowledge or dilemma, without realisation that statement was not true.

e.g. ‘She couldn’t keep them all. It’s kinder to kill them’, ‘She’s a horrible woman, she hates rabbits’, ‘She is evil, and the authorities should be called’.

*Party “Was there anything awkward or uncomfortable in this interaction? If so what was it?”*

2= ‘Yes’ + reference to the man making the situation awkward by assuming the man knew his son was gay and bringing it up in conversation. Some acknowledgment that one father was unaware and was now made aware.

1= ‘Yes’ + no further explanation; ‘Yes’ + reference to incorrect facts/intentions, or reference to attraction blooming/developing but no clear reference to the fact that the man let slip the young men were gay.

0= ‘No’

*Crying man “Why did the lady say "he is doing just fine"?”*

2 = reference to the woman’s use of sarcasm/irony/not being serious/being funny/ridicule/derision, the man is clearly not fine. (Answer MUST reference sarcasm/irony/not being serious/being funny/ridicule/derision). Simply ‘sarcasm’ is suffice for 2 points.

1= reference to the fact that the man is clearly not fine but without reference to sarcasm/irony/not being serious/being funny/ridicule/derision

e.g., ‘Because he is not fine’, ‘He is obviously not ok’

0 = reference to incorrect/irrelevant facts

e.g., ‘The man is fine, ‘She doesn’t want to get involved’, ‘Maybe she thinks he is actually ok’

*Burglar "Why does the burglar give himself up?”*

2 = reference to burglar’s ignorance of policeman’s true intention/knowledge state (Answer MUST have some reference to the thoughts of the policeman – i.e. the policeman had some knowledge/assumption/thought of the burglar’s wrong doing). ‘He thought he had been caught’ is not enough for 2 points without reference to the assumption being incorrect. (1 point). ‘Misunderstanding of policeman’s intentions’ is suffice for 2 points.

e.g., ‘He didn’t know the policeman just wanted to return his glove’, ‘He thought the policeman had seen him rob the shop’

1 = more general reference to burglar’s state of mind (e.g. ‘He thought he was being arrested’ , ‘He had a guilty conscience’) or outcome (e.g. ‘He thought the police might shoot otherwise’). Answer may reference thoughts of the policeman, but fail to link to policeman’s knowledge/assumption/thought of the burglar’s wrong doing.

e.g., ‘He thought he had been caught’

0 = ref to irrelevant facts/mental states

e.g., ‘He just wanted to come clean’, ‘He was tired of running’, ‘The police had his glove’

*Hat “Why does she say she loves the hat?”*

2= reference to white lie or wanting to spare the aunt’s feelings/cause offense/hurt aunt’s feelings. (Answer MUST include reference to the feelings of the aunt).

e.g., ‘To make her aunt feel good’, ‘To please her aunt’, ‘To make her aunt happy’ is suffice’

1= more general reference to trait (She’s a nice person; politeness), relationship (She likes her aunt), or social rules (It’s the socially appropriate thing to do).

0 = reference to irrelevant or incorrect facts/feelings

e.g., ‘She likes the hat’, ‘She wants to trick her’

*Spaghetti “When the mother said ‘that meal must have filled you up’ did she mean it? If not, why did she say it?”*

2**=** ‘No’ + reference to the mother’s use of sarcasm/irony/humour/derision/ridicule (Answer MUST reference sarcasm/irony/not being serious/being funny/ridicule/derision). ‘No’ + ‘Sarcasm’ is suffice for 2 points.

1**=** ‘No’ + no further explanation; No + reference to incorrect/irrelevant facts.

e.g., the boy didn’t eat much.

0**=** ‘Yes’

*Chocolate “Why does he look in the cupboard for the chocolates?”*

2= reference to the dad having lied/ being a liar/ he knows his dad is lying/trying to deceive him/trying to hide the chocolates from him/distrusts his father/ father is trying to deceive him/ father is trying to hide them from him etc.

1= reference to facts without explicit mention of lying.

e.g., ‘That’s where it really is’, ‘He knew it was in the cupboard’.

0= reference to general, story-nonspecific information.

e.g., ‘Chocolate is usually left in the cupboard/pantry’

*Christmas present “Why does Hannah say the present is just what she always wanted?”*

2 = reference to white lie or wanting to spare her parents’ feelings (Answer MUST reference to perception of parent’s feelings).

e.g., ‘So she doesn’t hurt their feelings’, ‘To please her parents’, ‘To make her parents happy’

1 = more general reference to trait (She’s well brought up) or emotion (She thinks it’s better than no present at all). She’s being polite/not rude/not being ungrateful.

0 = reference to irrelevant or incorrect facts/feelings

e.g., ‘She likes the books’, ‘She’s a hypocrite!’, ‘She secretly wanted encyclopaedias’

*Dinner “Is this true? Why does the mum say this?*”

2= ‘No’ + reference to the mother’s use of sarcasm/sheering or cutting remark/derision/irony/that she doesn’t really mean it, OR clear reference to sarcasm but have not circled ‘No’ (i.e. cannot have circled anything). Answer MUST reference sarcasm/irony/not being serious/being funny/ridicule/derision. ‘Sarcasm’ is enough for 2 points. No + ‘She was trying to point out that the daughter was being rude’ is not enough for 2 points (1 point).

1= ‘No’ + no further explanation; ‘No’+ reference to irrelevant facts/intentions (e.g. she thought her daughter was being nice and polite); OR incorrectly answered ‘Yes” but references manners, politeness, sarcasm etc. (Even if sarcasm is mentioned, if subject has circled ‘Yes’, a 2 cannot be scored).

e.g., ‘The mother wanted to point out the girl was being rude’, ‘The girl has bad manners’.

0= ‘Yes’

*Pregnancy “Was there anything awkward or uncomfortable in this interaction? If so what was it?”*

2= ‘Yes’ + reference to the girl mistakenly assuming that her friend was pregnant/the friend is not actually pregnant, possibly suggesting weight gain/that the friend is fat/blousy shirt. Yes + ‘she wasn’t pregnant’ is suffice for 2 points.

1= ‘Yes’ + no further explanation; ‘Yes’ + reference to incorrect/irrelevant facts

0= ‘No’

*Walking home “Why does she say this to the man?”*

2 = reference to her belief/expectation/presumption/incorrect assumption that he was going to mug her; she is paranoid. (Answer MUST reference her ignorance of his real intention). Use of the word paranoid implies ignorance of real intention. ‘She assumed he was going to mug her’ is not suffice for 2 points (1 point), unless they have stated the assumption was incorrect.

1 = reference to her trait (She’s a nervous person) or state (She’s scared/worried), or intention (So he wouldn’t hurt her) without suggestion that fear was unnecessary. ‘Because she thought he was going to mug her’ receives 1 point unless there is mention that she was incorrect.

0 = factually incorrect/irrelevant answers

e.g., ‘He would kill her if she didn’t give the purse!’

*Sausages “Why does the boy say this?”*

2= reference to intention to mislead, convince, persuasion, manipulation, guilt, making them feel sorry for him, lying to get what he wants/to get more. A mention that he is lying is suffice for 2 points.

1= reference to outcome (to get more), simple trait (greedy), or simple desire (wants more sausages).

0= reference to irrelevant facts

e.g., ‘Because his mum will have made him a lovely meal’, ‘He won’t be given dinner tonight’

*Alibi “Why does the man tell the policeman that his friend Pete is in the pub?”*

2= reference to the fact that the policeman will not believe him and hence look in the other place, to the man’s realisation that that’s what the policeman will do, or reference to a double bluff

1= reference to outcome (e.g. ‘To save his friend’) or simple lying (e.g. ‘To mislead them, to lie’)

0= reference to motivation that misses the point of double bluff. Any defeatist attitude/giving up (e.g. the policeman will find him anyway). To give him time to escape, but no further explanation.

e.g., ‘Because he was scared and wanted to tell the truth, didn’t want to be an accomplice’, ‘It’s the right thing to do’, ‘It’s better to give up his friend than lie to the police’, ‘he doesn’t want to cause any problems’.

*Cough “Is this true? Why does the father say this?”*

2= ‘No’ + reference to what he says as being a figure of speech/slang/colloquialism/common saying/expression that people use/well-known saying/analogy

1= ‘No’ + no further explanation; ‘No’ + reference to irrelevant facts/intentions. ‘yes’ + a correct response receives 1 point.

0= ‘Yes’

*Dog “Is this true? Why does Hannah say this?”*

2= ‘No’ + reference to Hannah making a joke/being silly/being sarcastic; friendly exaggeration. (Answer MUST make reference to joke/idiom/sarcasm/irony/banter/humour)

1= ‘No’ + no further explanation; ‘No’ + reference to irrelevant facts/intentions general (e.g. ‘She thought the dog was an elephant); ‘No’ + reference to the dog’s size (e.g. ‘The dog is big’, ‘She was expecting a smaller dog’)

0 = ‘Yes’

*Banana “Is this true? Why does the boy say this?”*

2= ‘No’ + reference to the boy pretending the banana is a phone, banana being shaped like a phone. MUST use word pretend/make believe/imagination/imaginative/imagine/imagining. ‘No’ + ‘He was joking’ is not suffice for 2 points (1 point). ‘No’ + ‘The banana is a similar shape to a phone’ is not suffice for 2 points (1 point).

1= ‘No’ + no further explanation; ‘No’ +reference to irrelevant facts/intentions

e.g., ‘He was trying to impress the girl’.

0= ’Yes’

*Vase “Is this true? Why did she say it was the dog?”*

2= ‘No’ +reference to the fact that the girl was lying, that saying the dog did it would shift the blame off her and avoid her getting in trouble; punishment avoidance. (Answer MUST reference shifting the blame)

e.g., ‘She didn’t want to get in trouble’

1= ‘No’+ no further explanation; ‘No’ + reference to incorrect/irrelevant facts.

e.g., ‘She is a brat’

0 = ‘Yes’

*Physical items*

*Light bulbs “Why does he buy the pack of 10?”*

2= reference to saving money (since multipacks are cheaper). May also, but needn’t mention convenience of having more or future need for more than one bulb.

e.g., ‘Better value’, ‘Cheaper in bulk’, ‘Saves money that way’

1= reference to convenience of having more, or future need for more than one bulb. No mention of saving money or better value.

e.g. ‘So he won’t have to keep going out to the store’, ‘In case one blows’, ‘He will need more later’.

0= reference to irrelevant or incorrect facts or references to characteristics of salesman

e.g.’ He likes that brand the best’. ‘He needs a whole lot of bulbs’, ‘The salesman was charismatic’, ‘It was a good sales pitch’, ‘He was a con artist’.

*Swimming (competition) “Why did Harry win?”*

2= reference to the race being in the ocean/waves/surf/beach not a swimming pool, that Harry is faster in the ocean/waves/surf/beach (Answer MUST reference ocean/waves/beach/surf).

e.g., ‘He is better in the ocean’, ‘He has more experience in the surf/ocean’.

1 = N/A

0 = Reference to irrelevant or incorrect information

e.g., Harry is a better swimmer, older, male, psychologically strong/he tricked her/had a head start/can run faster).

*(Lost) glasses “Why is the post office the most likely place to look?”*

2= reference to post office being the place she would most likely use her glasses (to read/write/fill out forms); wouldn’t need at gym/flower shop. Simply ‘It was the most likely place she would have left them/would need to use them’ is suffice for 2 points.

1= general reference to post office being where she left them

0 = reference to irrelevant or incorrect factors

e.g. ‘That was the last or first place she went’, ‘He decided to go there first as it was the closest’.

*Car “Why does he accept the dealer’s offer to pay in monthly instalments?”*

2= reference to relative gain from leaving money in the bank due to greater interest gained on savings than spent on monthly instalment payments (exact figures not necessary, but must suggest interest is better/different/more etc.).

e.g., ‘Because his bank pays better interest than the dealer charges’, ‘8-5 = 3% profit interest’

1= general reference to saving money that way, or it being the sensible thing to do. General reference to interest rates but without specific reference to saving money based on interest rates

e.g., ‘Because of the interest rates’, ‘He thought it was the smart thing to do’

0= reference to irrelevant or incorrect factors.

e.g. ‘He can’t afford the whole thing’, ‘He wants to keep some money in the bank to pay bills’.

*Leg injury “Why does she need an x-ray?”*

2= reference to the possibility that she has fractured/broken/cracked/split her leg. Seems to have understood that she may have caused further injury to her leg and there is a need to assess this damage.

e.g., ’They want to see if she has broken anything’, ‘She may have fractured her hip’ ‘The possibility of a fracture’, ‘She may have damaged her bone’, ‘To check for internal damage’

1= reference to general aim. Is not specific to checking the leg for further injury.

e.g., ‘To see what’s wrong’, ‘Because of her fall’, ‘Because her leg is swollen’

0= reference to irrelevant or incorrect factors

e.g., ‘That’s what doctors do’, ‘It’s standard procedure’

*Librarian “Why did Mrs Simpson put the book in a special room?”*

2= reference to delicate condition of the book due to age or value; Any reference to preservation/protection/keeping it safe is suffice; May reference temperature control in the room.

e.g., ‘It may be old and requires special handling’, ‘To protect it’

1=general reference to special status of the book, not further explained

e.g., ‘The book is old’, ‘It is special’

0= reference to other motivations not warranted by the story

e.g., ‘So she would always know where to find it/for her own convenience’, ‘The book contains plant specimens’.

*Burglar alarm “Why did the alarm go off?”*

**2**= reference to an animal, which the burglar disturbed, setting off the alarm (any type of animal is okay e.g. cat, mouse, rat, or reference to something small).

e.g., ‘The motion sensor was triggered by a mouse’, ‘The cat ran through the beam’.

1**=** reference to burglar setting off the alarm (e.g. being startled by the animal and setting off the sensor).

e.g., ‘The burglar accidentally set it off after he saw the mouse’, ‘He got scared and fell over, tripping the beam’.

0**=** reference to irrelevant or incorrect facts.

e.g., ‘The animal’s screech set off the alarm’, ‘The burglar was scared’.

*Mayonnaise “Why does she suggest to make meringues?”*

2= reference to the egg whites left from making mayonnaise used up in making meringues. If yolk is mistaken for white, a score of 2 can still be awarded.

e.g., ‘Because meringues are made of egg whites’, ‘To use the egg whites’, ‘To not waste the whites’

1= general reference to not wasting anything, not further explained

0 = reference to other irrelevant motivations

e.g., ‘To show off’, ‘To have more choice’, ‘It would make a mess with the whites’

Figure 1

*Scree Plot for PCA of A-ToM 12 Items*


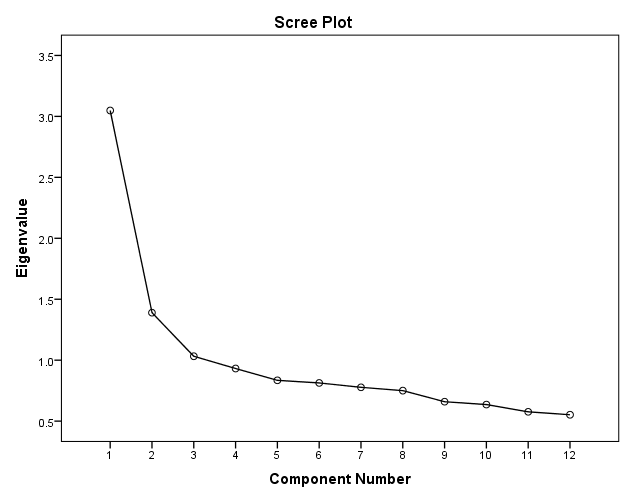


Table 3

*Descriptive statistics for the Interpersonal Reactivity Index and the mini-SPIN for the ASD and non-ASD control sample*.

|  | Group | |
| --- | --- | --- |
| Descriptive Statistics | ASD  (*N* = 163) | Non-ASD  (*N* = 80) |
| Interpersonal Reactivity Index |  |  |
| Empathic concern |  |  |
| Mean (& SD) | 16.1 (4.7) | 18.9 (3.9) |
| 95% CIs | [15.4, 16.8] | [18.1, 19.8] |
| Cohen’s *d (& 95% CIs)* | 0.63 [0.36, 0.91] | |
| Perspective taking |  |  |
| Mean (& SD) | 14.1 (5.4) | 19.0 (4.7) |
| 95% CIs | [13.2, 14.9] | [17.9, 20.0] |
| Cohen’s *d* | 0.95 [0.67, 1.23] | |
| Personal distress |  |  |
| Mean (& SD) | 13.4 (5.2) | 10.8 (5.0) |
| 95% CIs | [12.6, 14.2] | [9.7, 11.9] |
| Cohen’s *d (& 95% CIs)* | 0.50 [0.23, 0.78] | |
| mini-SPIN |  |  |
| Mean (& SD) | 6.6 (2.8) | 4.5 (2.7) |
| 95% CIs | [6.2, 7.0] | [3.9, 5.1] |
| Cohen’s *d (& 95% CIs)* | 0.77 [0.49, 1.04] | |

Table 4

*Inter-correlations of A-ToM scales with other ToM measures and Mini SPIN and IRI scales for ASD sample*

| Measure | 1. | 2. | 3. | 4. | 5. | 6. | 7. | 8. | 9. | 10. |
| --- | --- | --- | --- | --- | --- | --- | --- | --- | --- | --- |
| 1. A-ToM social | - | .42** | .58** | .53** | .22** | .34** | .09 | .13 | -.01 | .12 |
| 2. A-ToM physical |  | - | .37** | .61** | .14 | .29** | -.08 | .07 | -.05 | .01 |
| 3. SS social |  |  | - | .62** | .18* | .27** | .14 | .06 | .01 | .06 |
| 4. SS physical |  |  |  | - | .16* | .31** | .06 | .09 | -.01 | .01 |
| 5. Frith-Happé mental |  |  |  |  | - | .79** | .12 | .01 | -.00 | .09 |
| 6. Frith-Happé feeling |  |  |  |  |  | - | .06 | .03 | .08 | .03 |
| 7. Mini SPIN |  |  |  |  |  |  | - | .11 | .38** | -.10 |
| 8. IRI Empathic Concern |  |  |  |  |  |  |  | - | .37** | .36** |
| 9. IRI Personal Distress |  |  |  |  |  |  |  |  | - | -.14 |
| 10.IRI Perspective Taking |  |  |  |  |  |  |  |  |  | - |

**p* < .05, ***p* < .01 (2-tailed)

*Inter-correlations of A-ToM scales with other ToM measures and Mini SPIN and IRI scales for non-ASD sample*

| Measure | 1. | 2. | 3. | 4. | 5. | 6. | 7. | 8. | 9. | 10. |
| --- | --- | --- | --- | --- | --- | --- | --- | --- | --- | --- |
| 1. A-ToM social | - | .27^*^ | .49^**^ | .41^**^ | .17 | .15 | .03 | .16 | .11 | -.04 |
| 2. A-ToM physical |  | - | .24^*^ | .42^**^ | .09 | .08 | -.28^*^ | -.00 | .07 | -.04 |
| 3. SS social |  |  | - | .58^**^ | .06 | .13 | .04 | -.01 | -.10 | -.05 |
| 4. SS physical |  |  |  | - | .09 | .12 | .04 | .13 | -.02 | .08 |
| 5. Frith-Happé mental |  |  |  |  | - | .80^**^ | .05 | -.01 | .25^*^ | -.12 |
| 6. Frith-Happé feeling |  |  |  |  |  | - | .16 | -.02 | .19 | -.18 |
| 7. Mini SPIN |  |  |  |  |  |  | - | .03 | .35^**^ | -.03 |
| 8. IRI Empathic Concern |  |  |  |  |  |  |  | - | .26^*^ | .50^**^ |
| 9. IRI Personal Distress |  |  |  |  |  |  |  |  | - | -.13 |
| 10.IRI Perspective Taking |  |  |  |  |  |  |  |  |  | - |

**p* < .05, ***p* < .01 (2-tailed)

*Inter-correlations of A-ToM scales with other ToM measures and Mini SPIN and IRI scales for ASD and non-ASD samples combined*

| Measure | 1. | 2. | 3. | 4. | 5. | 6. | 7. | 8. | 9. | 10. |
| --- | --- | --- | --- | --- | --- | --- | --- | --- | --- | --- |
| 1. A-ToM social | - | .39^**^ | .60^**^ | .54^**^ | .25^**^ | .33^**^ | -.03 | .20^**^ | -.05 | .19^**^ |
| 2. A-ToM physical |  | - | .35^**^ | .56^**^ | .14^*^ | .24^**^ | -.16^*^ | .08 | -.04 | .04 |
| 3. SS social |  |  | - | .64^**^ | .19^**^ | .28^**^ | -.01 | .14^*^ | -.09 | .16^*^ |
| 4. SS physical |  |  |  | - | .18^**^ | .29^**^ | -.03 | .17^**^ | -.07 | .12 |
| 5. Frith-Happé mental |  |  |  |  | - | .79^**^ | .03 | .05 | .03 | .10 |
| 6. Frith-Happé feeling |  |  |  |  |  | - | .02 | .07 | .07 | .05 |
| 7. Mini SPIN |  |  |  |  |  |  | - | -.02 | .42^**^ | -.21^**^ |
| 8. IRI Empathic Concern |  |  |  |  |  |  |  | - | .24^**^ | .46^**^ |
| 9. IRI Personal Distress |  |  |  |  |  |  |  |  | - | -.22^**^ |
| 10.IRI Perspective Taking |  |  |  |  |  |  |  |  |  | - |

**p* < .05, ***p* < .01 (2-tailed)
